# Transforming Patient‐Reported Outcome Measurement With Digital Health Technology

**DOI:** 10.1111/jep.70107

**Published:** 2025-04-29

**Authors:** Liam Jackman, Rakhshan Kamran

**Affiliations:** ^1^ Temerty Faculty of Medicine University of Toronto Toronto Canada; ^2^ Nuffield Department of Orthopaedics, Rheumatology and Musculoskeletal Sciences University of Oxford Oxford UK

**Keywords:** health services research, patient‐centred care, person‐centred medicine

## Abstract

Healthcare is shifting from a provider‐centric to a patient‐centric model, emphasizing the integration of patient‐reported outcome measures (PROMs) into routine practice. PROMs enhance shared decision‐making and provide valuable insights into patient well‐being, yet their widespread implementation is hindered by logistical challenges, time constraints, and infrastructure limitations. Digital health solutions offer a promising approach to overcoming these barriers by streamlining PROM administration, improving accessibility, and optimizing clinical integration. This article explores the transition from paper‐based to digital PROM administration, the advantages of computerized adaptive testing (CAT), and the broader considerations necessary to ensure effective implementation. By leveraging digital tools and informatics strategies, healthcare systems can facilitate the meaningful adoption of PROMs to improve patient‐centred care. This article can be used to advance PROM implementation across various clinical settings.

## 1 Introduction

Healthcare is undergoing a paradigm shift from provider‐driven decision‐making to patient‐centred models that prioritize individual experiences and perspectives [1, 2]. Patient‐reported outcome measures (PROMs) serve as a critical tool in this transformation by capturing data directly from patients regarding their symptoms, functional status, and quality of life [2, 3, 4]. Traditionally, healthcare outcomes have been assessed using objective clinical indicators such as hospital readmission rates, and physiological metrics like blood pressure or cholesterol levels [2, 3]. While these metrics provide important information, they often fail to capture the patient's perspective and lived experience [2]. PROMs fill this gap by capturing subjective health concepts that matter most to patients [4, 5, 6]. By incorporating PROMs into clinical practice, healthcare professionals can facilitate shared decision‐making, monitor treatment effectiveness, and improve healthcare delivery [5, 6]. However, despite their benefits, the integration of PROMs into routine care remains inconsistent due to significant barriers, including infrastructure limitations, response burden, data integration challenges, and clinician engagement issues [7]. Many healthcare institutions still rely on paper‐based PROM administration, which is time‐intensive and prone to errors, further limiting their clinical utility [8, 9, 10, 11]. Additionally, some PROMs are lengthy and complex, making them difficult to complete in time‐constrained clinical settings [11]. To fully realize their potential, healthcare systems must adopt innovative digital health strategies that enhance PROM accessibility, streamline implementation, and optimize data utilization within clinical workflows.

## 2 Challenges in PROM Implementation

One of the primary barriers to PROM implementation is the continued reliance on paper‐based administration [8]. Many healthcare institutions still use printed forms that require manual completion, scoring, and then transcription into electronic medical records. This method is inefficient, prone to errors, and contributes to a significant administrative burden for both clinicians and patients. Additionally, scanned PROM responses often remain buried in electronic medical records without structured data integration, limiting their potential impact on clinical decision‐making [9]. The absence of automated data capture further delays analysis and reduces the timeliness of insights that could inform patient care.

Time constraints also pose a considerable challenge, particularly in clinical settings where appointment times are limited. Many PROMs are lengthy and complex, making them difficult to administer within the confines of a standard patient visit [11]. As a result, patients may not complete them fully or accurately, and clinicians may struggle to interpret responses in real‐time [11, 12, 13]. While remote completion of PROMs outside clinical encounters is a potential solution, adherence rates vary, particularly among populations with limited health literacy, cognitive impairments, or reduced access to digital technologies [14, 15, 16, 17, 18, 19, 20].

Clinician engagement is another critical factor influencing PROM implementation. Many clinicians express concerns that integrating PROMs into their workflow will add to their workload, rather than streamline care [12, 13]. Some also worry about data overload, wondering whether they will have the time and resources to meaningfully interpret and act on patient‐reported data [2, 9]. Additionally, variances in clinician training on PROM utilization and a lack of standardized frameworks for integrating PROM data into clinical workflows contribute to hesitancy in adoption [12, 13, 14].

Another significant barrier is the digital divide. Although digital PROM solutions offer numerous benefits, not all patients have equal access to technology. Individuals from lower socioeconomic backgrounds, older adults, and those with limited digital literacy may struggle to engage with electronic PROMs (ePROMs), potentially exacerbating healthcare disparities [20]. Addressing this challenge requires a concerted effort to ensure that digital PROM platforms are user‐friendly, accessible, and inclusive.

Additionally, marginalized populations, including transgender and gender‐diverse individuals, may experience barriers beyond the digital divide [21, 22]. Mistrust in healthcare systems, concerns about the inclusivity of PROM questions, and discomfort with providing personal health data can discourage participation [23, 24, 25, 26]. Many standard PROMs fail to reflect the unique experiences and healthcare needs of marginalized populations, leading to data which may not accurately capture their health concerns. PROMs that include accurate and inclusive language and allow for self‐identification are necessary to ensure equitable implementation [25, 26]. Implementing PROMs in settings with marginalized populations requires additional considerations such as staff training on inclusive data collection, and ensuring that patient‐reported outcomes are used to improve rather than pathologize patient experiences.

## 3 Digital Health as a Solution

Digital health technologies present a powerful solution to many of the barriers associated with PROM implementation [27, 28, 29]. Transitioning from paper‐based to ePROMs streamlines data collection, enhances accessibility, and improves integration into electronic health records (EHRs) [27, 28, 29]. ePROM platforms allow patients to complete assessments remotely at their convenience, reducing the burden on clinic staff and ensuring that data is available ahead of consultations. Moreover, the automation of data capture minimizes transcription errors, facilitates real‐time analysis, and enables clinicians to incorporate patient‐reported insights into their decision‐making processes more effectively [30, 31, 32, 33, 34, 35, 36, 37].

One promising innovation in digital PROM administration is computerized adaptive testing (CAT) [37]. Unlike traditional static PROMs, CAT employs algorithms that tailor question selection based on patient responses, eliminating unnecessary or redundant items. This approach reduces the length of assessments while maintaining measurement accuracy, improving patient engagement and response rates. CAT‐based PROMs can lead to greater efficiency, reducing patient burden without compromising data quality [38, 39].

Interoperability is another critical component of successful digital PROM integration. By leveraging standardized frameworks such as HL7 FHIR and SNOMED CT, PROM data can be seamlessly exchanged across healthcare systems, supporting continuity of care and data‐driven decision‐making. Additionally, predictive analytics and artificial intelligence (AI) can further enhance PROM utility by identifying trends, stratifying risk, and generating alerts for clinicians when patient‐reported data indicates potential deterioration in health status. These capabilities support proactive intervention and more personalized care planning.

## 4 Addressing Implementation Barriers

Despite advantages of digital PROMs, their successful implementation requires thoughtful planning and investment in infrastructure, security, and user engagement [35]. Healthcare organizations must allocate resources to develop user‐friendly digital platforms, ensure interoperability with existing EHR systems, and provide training for clinicians and patients. While initial costs may be high, long‐term benefits include improved efficiency, reduced administrative burden, and enhanced patient outcomes [40, 41, 42, 43].

Security and privacy concerns are highly important when implementing digital PROMs. Compliance with regulations such as HIPAA and GDPR is essential to protect patient data and maintain trust [36]. Encryption protocols, secure storage solutions, and clear data governance policies should be prioritized to mitigate risks associated with data breaches and unauthorized access [36, 37]. Transparent communication about how PROM data will be used and safeguarded can further enhance patient and clinician confidence in digital PROM adoption.

Clinician engagement strategies must also be prioritized to ensure successful PROM implementation. Providing targeted training, integrating PROM insights into clinical decision‐support tools, and designing intuitive interfaces can improve clinician confidence and uptake. The Technology Acceptance Model (TAM) suggests that technology adoption is driven by perceived ease of use and usefulness, reinforcing the need for well‐designed, efficient, and seamlessly integrated digital PROM platforms [37]. Additionally, incorporating patient‐centred design principles and seeking feedback from diverse patient populations can enhance usability and engagement [44, 45, 46, 47, 48, 49, 50, 51, 52].

## 5 Conclusion

The integration of digital health solutions represents a transformative opportunity to overcome longstanding barriers to PROM implementation. By transitioning to ePROMs administration, leveraging adaptive testing methodologies, and ensuring seamless interoperability, healthcare systems can unlock the full potential of patient‐reported outcomes to enhance clinical decision‐making and patient‐centred care. However, addressing challenges related to infrastructure, security, and user adoption remains critical to ensuring sustainable implementation. Future research should explore the integration of ePROM solutions and predictive analytics to further optimize patient monitoring and personalized care strategies. With strategic investment and commitment to digital transformation, PROMs can become a cornerstone of modern, patient‐centred healthcare.

## Author Contributions

Liam Jackman and Rakhshan Kamran conceptualized this paper. Liam Jackman led the writing of the manuscript. Rakhshan Kamran was involved with the critical revision of the manuscript. All co‐authors approve of the submission.

## Ethics Statement

The authors have nothing to report.

### 1 Conflicts of Interest

The authors declare no conflicts of interest.

## Data Availability

The authors have nothing to report. All information and materials in the manuscript are original.
